# Prediction of posthepatectomy liver failure using transient elastography in patients with hepatitis B related hepatocellular carcinoma

**DOI:** 10.1186/s12876-017-0732-4

**Published:** 2017-12-29

**Authors:** Jie-wen Lei, Xiao-yu Ji, Jun-feng Hong, Wan-bin Li, Yan Chen, Yan Pan, Jia Guo

**Affiliations:** 1 Department of Ultrasound, Eastern Hepatobiliary Surgery Hospital (EHBH), Second Military Medical University, Shanghai, China; 2 People’s Liberation Army Military Academy, Second Military Medical University, Shanghai, China; 3 Department of Ultrasound, FuZhou General Hospital (Dongfang Hospital), Xiamen University, Fuzhou, Fujian China; 4 Department of Ultrasound, Shanghai First People’s Hospital, Shanghai, China; 5 Department of Ultrasound, Yuhuangding Hospital, Yantai, Shandong China

**Keywords:** Hepatocellular carcinoma, Hepatectomy, Posthepatectomy liver failure, Liver stiffness measurement, Hepatitis B

## Abstract

**Background:**

It is essential to accurately predict Postoperative liver failure (PHLF) which is a life-threatening complication. Liver hardness measurement (LSM) is widely used in non-invasive assessment of liver fibrosis. The aims of this study were to explore the application of preoperative liver stiffness measurements (LSM) by transient elastography in predicting postoperative liver failure (PHLF) in patients with hepatitis B related hepatocellular carcinoma.

**Methods:**

The study included 247 consecutive patients with hepatitis B related hepatocellular carcinoma who underwent hepatectomy between May 2015 and September 2015. Detailed preoperative examinations including LSM were performed before hepatectomy. The endpoint was the development of PHLF.

**Results:**

All of the patients had chronic hepatitis B defined as the presence of hepatitis B surface antigen (HBsAg) for more than 6 months and 76 (30.8%) had cirrhosis. PHLF occurred in 37 (14.98%) patients. Preoperative LSM (odds ratio, OR, 1.21; 95% confidence interval, 95% CI: 1.13–1.29; *P* < 0.001) and international normalized ratio (INR) (OR, 1.07; 95% CI: 1.01–1.12; *P* < 0.05) were revealed to be independent risk factors for PHLF, and a new model was defined as LSM-INR index (LSM-INR index = 0.191*LSM + 6.317*INR-11.154). The optimal cutoff values of LSM and LSM-INR index for predicting PHLF were 14 kPa (AUC 0.86, 95% CI: 0.811–0.901, *P* < 0.001) and −1.92 (AUC 0.87, 95% CI: 0.822–0.909, *P* < 0.001), respectively.

**Conclusions:**

LSM can be helpful for surgeons to make therapeutic decisions in patients with hepatitis B related hepatocellular carcinoma.

## Background

Hepatocellular carcinoma (HCC) is one of the common malignant tumors worldwide. Surgical resection is the most effective treatment for patients with localized HCC [1, 2]. However, postoperative liver failure (PHLF) is a life-threatening complication and intrinsic risk of mortality [3]. It is not only correlated with the volumes of liver resection, but also the insufficient function of hepatic reserve (FHR) [4].

More than 80% of HCCs arise in patients with hepatic fibrosis or cirrhosis [5], which has a very important impact on liver function [4]. Hence, we attempted to determine whether FHR can be indirectly predicted by assessing the degree of liver fibrosis.

LSM is a technology involving obtaining the liver instantaneous elastic modulus to estimate the degree of liver fibrosis by transient elastography (TE), an easy and noninvasive method with high accuracy [6–8].

Recently, several studies reported that liver stiffness is associated with posthepatectomy outcomes [10–12]. However, baseline characteristics of patients differed greatly among these studies, and few of the studies focused on PHLF. Unlike those in the western countries, about 80% HCC patients in China had hepatitis B virus infection [13].

### Aim of the study

In this study, we aimed to assess the usefulness of liver stiffness measured by transient elastography Fibro Touch® (Wuxi HISKY Medical Technologies Co., Ltd. Beijing, China) for predicting PHLF in patients with hepatitis B-related hepatocellular carcinoma.

## Methods

### Patients

This study was approved by the Institutional Ethics Committee of the Eastern Hepatobiliary Surgery Hospital (EHBH).

HCC patients who underwent liver resection at EHBH were prospectively recruited between May 2015 and September 2015. The exclusion criteria were as follows: (i) patients with hepatolithiasis or patients who will receive hepatectomy because of intrahepatic cholangiocarcinoma (ICC) or hepatic maligancies other than HCC; (ii) patients with cirrhosis due to schistosomiasis, alcoholic liver disease or non-alcoholic fatty liver disease (NAFLD); (iii) patients undergoing preoperative transhepatic arterial chem otherapy and embolization (TACE).

### Transient elastography

All patients fasted for at least 6 h before receiving LSM examination by transient elastography FibroTouch®. The examination was performed by two trained and certified operators who were blinded to the patients’ clinical data, according to the operation manual and the Liver Stiffness Study Group “Elastica” of the Italian Association for the Study of the Liver [14]. LSM was expressed in kiloPascals (kPa) and was considered reliable only if 10 successful measurements were obtained, with an IQR/median of LSM of < 30% and a success rate of > 60% [15].

### Liver surgery

During surgery, right costal margin incision was chosen and the fluid infusion was minimal to keep central venous pressure lower than 5 mmHg to reduce bleeding from hepatic veins [16, 17]. Intraoperative ultrasound (US) was performed systematically to detect the presence of any additional nodules not detected preoperatively. Major hepatectomy was defined as removal of 3 or more Couinaud segments [18, 19]. Diuretics and Ampicillin were used for routine postoperative care.

PHLF was defined as the presence of at least one of the following variables: 1) occurrence of refractory ascites causing a delay in the removal of surgical drainages and/or postoperative drainage exceeding 500 ml/day, a continuous elevation of total serum bilirubin concentration (≥60umol/l) beyond postoperative day 7; 2) alteration of coagulation factors requiring fresh frozen plasma infusion with an International Normalized Ratio (INR) of more than 1.50 [20]. The endpoint of this study is the presence of PHLF.

### Statistical analysis

Continuous variables were expressed as the mean and standard deviation. Differences between the subgroups were compared by *t*-test or Mann-Whitney U test. Categorical variables were compared using χ^2^ test with Yates’ correction or Fisher’s exact test. Factors with significant impact on PHLF upon univariate analysis were explored with multivariate forward logistic regression as hypothetical independent predictors of PHLF. A significance level of 0.05 was used in all analyses. The prognostic value of PHLF prediction model and the LSM only were assessed using receiver operating characteristic (ROC) curve analysis (MedCalc Software bvba, Ostend, Belgium). The area under the ROC curve (AUC), the sensitivity, the specificity, the positive and negative predictive values, and the positive and negative likelihood ratio for cutoff values were obtained.

Data analysis was performed using SPSS, version 19.0 for Windows (SPSS, Inc., Chicago, IL) and R software 2.10.1 (R Foundation for Statistical Computing, Vienna, Austria; www.r-project.org). All reported *p* values were two-sided, and *p* < 0.05 was considered to be statistically significant.

## Results

### Characteristics of the study population

The demographic and clinicopathologic characteristics of these patients were shown in Table 1.

**Table 1:** Baseline characteristics of patients (*N* = 247)

Variables	n (%), mean ± SD, or median (range)
Age (years)	53.3 ± 10.3
Gender (Male /Female)	213/34(86%/14%)
BMI (kg /m2)	29.9 ± 16.5
Complications^a^	43(17%)
Cirrhosis (yes /no)	76/171(30.8%/69.2%)
White blood cell (10^9^/l)	5.2 ± 2.0
Hemoglobin (g/l)	143.9 ± 14.1
Platelet Count (10^9^/l)	145(41–466)
Child–Pugh class A	247
Total bilirubin (umol/l)	16.0 ± 11.8
Albumin (g/l)	41.7 ± 3.3
Prealbumin (mg/l)	238.6 ± 62.6
Alanine transaminase (u/l)	39.2 ± 34.2
Aspartate aminotransferase (u/l)	36.99 ± 31.3
Gamma-glutamyl transpeptidase (u/l)	47(11–866)
Alkaline phosphatase (u/l)	81(34–250)
PT(s)	11.5(9.4–15.4)
INR	1.0(0.8–1.3)-
APTT(s)	27.2(16.7–52.9)
LSM(Kpa)	12.7(3.8–38.5)
Intraoperative blood infusion (yes / no)	19/228(8%/92%)
Portal vein occlusion (yes / no)	194/53(79%/21%)
Esophageal varices (yes / no)	18/229(7.3%/92.7%)
Tumor capsule (yes / no)	174/73(70%/30%)
Number of tumors (Single / multiple)	217/30(88%/12%)
Anti-viral medication(Positive / negative)	71/176(28.7%/71.2%)
HBV DNA levels (>1.0E + 04iu/ml/<1.0E + 04iu/ml)	73/174(30%/70%)
Major hepatectomy	40 (16%)
Median main tumor size(cm)	5.02 ± 3.18
Tumor location (right lobe /left lobe/ both)	158/79/10(64%/32%/4%)

^a^Complications include one or more of the following: hypertension, heart disease (myocardial ischemia, cardiomyopathy, arrhythmia), chronic obstructive pulmonary disease, diabetes, etc.

All the patients (213 men and 34 women), with a mean age of 53.27 ± 10.33 years, had postive HBsAg lasting for > 6 months. All of them were Child-Pugh class A, and a small proportion had complications (17%). As shown by the pathological results, 76 (31%) patients had cirrhosis and 18 (7%) had esophageal varices consistent with gastroscopy results. 37 (14.98%) patients developed PHLF postoperatively, and they had a significantly higher preoperative mean LSM (21.4 ± 6.3 kPa) than those without PHLF (12.7 ± 5.7 kPa, *P* < 0.001).

### Independent predictors for PHLF in HCC patients

Univariate and multivariate analysis were used for analyzing the potential influencing factors associated with PHLF, and the results were reported in Table 2.

**Table 2:** Univariate analysis and multivariate linear regression analysis of the variables associated with PHLF

Variables	Univariate analysis			Multivariate analysis		
	PLF(+)(*n* = 37)	PLF(−)(*n* = 210)	*p*	Exp(B)	OR95%CI	*p*
Age (years)	54.9 ± 10.9	53.0 ± 10.2	0.286			
Gender (Male /Female)	33/4 (89%/11%)	180/30 (86%/14%)	0.572			
BMI (kg /m2)	23.7 ± 3.1	23.3 ± 3.0	0.423			
White blood cell (10^9^/l)	4.9 ± 1.5	5.3 ± 2.1	0.275			
Hemoglobin (g/l)	144.2 ± 12.3	143.8 ± 14.4	0.881			
Platelet Count (10^9^/l)	139.0 ± 79.9	155.3 ± 56.9	0.037			
Total bilirubin (umol/l)	19.1 ± 8.8	15.4 ± 12.2	0.079			
Albumin (g/l)	41.0 ± 3.6	41.9 ± 3.2	0.123			
Prealbumin (mg/l)	218.2 ± 67.9	242.2 ± 61.0	0.032			
Alanine transaminase (u/l)	43.2 ± 31.3	38.5 ± 34.7	0.449			
Aspartate aminotransferase (u/l)	41.2 ± 23	36.1 ± 32.4	0.366			
Gamma-glutamyl transpeptidase (u/l)	104.8 ± 148.6	68.6 ± 69.2	0.027			
Alkaline phosphatase (u/l)	100.0 ± 47.4	83.2 ± 23.3	0.173			
PT(s)	12.1 ± 1.2	11.6 ± 0.8	0.005			
INR	1.0 ± 0.1	1.0 ± 0.1	0.005	1.065	1.014–1.119	0.013
APTT(s)	28.9 ± 5.9	27.1 ± 5.2	0.067			
LSM(kpa)	21.4 ± 6.3	12.7 ± 5.7	<0.001	1.211	1.134–1.293	<0.001
Intraoperative blood infusion (yes /no)	7/30 (18.9%/81.1%)	12/198 (5.7%/94.3%)	0.005			
Portal vein occlusion (yes/no)	24/13 (64.9%/35.1%)	160/50 (76.2%/23.8%)	0.145			
Number of tumors (Single/multiple)	32/5 (86.5%/13.5%)	185/25 (88.1%/11.9%)	0.782			
Tumor capsule (yes/no)	24/13 (64.9%/35.1%)	150/60 (71.4%/28.6%)	0.420			
HBV DNA levels (>1.0E + 04iu/ml/<1.0E + 04iu/ml)	13/24 (35.1%/64.9%)	60/150 (28.6%/71.4%)	0.422			
Cirrhosis (yes/no)	22/15 (59.5%/40.5%)	54/156 (27.7%/74.3%)	<0. 001			
Esophageal varices (yes/no)	7/30 (18.9%/81.1%)	11/199 (5.2%/94.8%)	0.003			
Major hepatectomy	8 (22%)	32 (15%)	0.331			
Median main tumor size (cm)	5.6 ± 4.0	4.9 ± 3	0.687			
Tumor location (right lobe/left lobe/both)	29/6/2 (78.4%/16.2%/5.4%)	129/73/8 (61.4%/34.8%/3.8%)	0.082			

Univariate analysis revealed that the factors including platelet count (*p* = 0.037), prealbumin (*p* = 0.032), gamma-glutamyl transpeptidase (*p* = 0.027), prothrombin time (*p* = 0.005) and INR (*p* = 0.005), LSM (*p* < 0.001), the use of intraoperative blood transfusions (*p* = 0.005), the presence of cirrhosis (*p* < 0.001) and esophageal varices (*p* = 0.003) were significant predictors for PHLF.

Multivariate analysis showed that only LSM (odds ratio, OR, 1.2; 95% confidence interval, 95% CI, 1.134–1.293, *P* < 0.001) and INR (OR, 1.1; 95% CI, 1.014–1.119, *p* < 0.05) remained in a binary logistic regression model, and revealed they were independent risk factors for PHLF. Meanwhile, a new algorithm was defined for predicting PHLF: the LSM-INR index = 0.191*LSM + 6.317*INR-11.154.

### Diagnostic performance of LSM for predicting PHLF

**Fig. 1:**
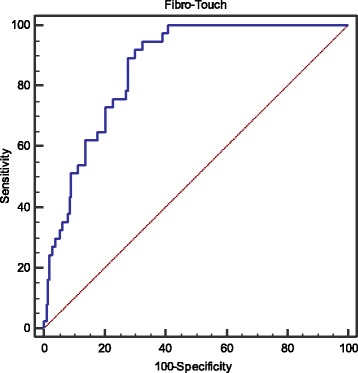
ROC analysis of liver stiffness measurement for predicting PHLF

The diagnostic performance and corresponding ROC curves of LSM are shown in Fig. 1. The optimal cutoff value of LSM is 14 kPa for predicting PHLF [AUC 0.860 95% CI: 0.811–0.901, *p* < 0.001; sensitivity (Se) 94.6%, specificity (Sp) 67.6%, positive predictive values (PPV) 34%, negative predictive values (NPV) 98.6%, positive likelihood ratio (LR^+^) 2.9, negative likelihood ratio (LR^−^) 0.1].

**Fig. 2:**
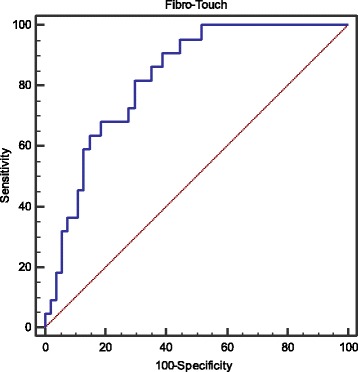
ROC analysis of liver stiffness measurement with only cirrhotic patients for predicting PHLF

When considering cirrhotic patients only, ROC curve analysis identified the best cutoff value of LSM is 17.0 kPa for predicting PHLF (AUC 0.825, 95% CI: 0.721–0.903, *p* < 0.001; Se 81.8%, Sp 70.4%, PPV 52.9%, NPV 90.5%, LR^+^ 2.86, LR^−^ 0.3) (Fig. 2).

**Fig. 3:**
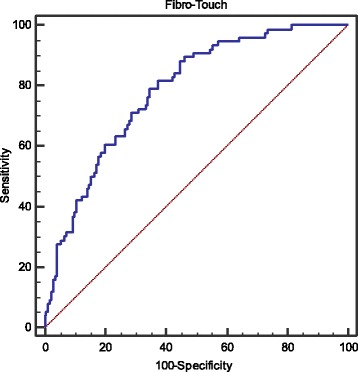
ROC analysis of liver stiffness measurement for predicting cirrhosis

Meanwhile, the optimal cutoff value of LSM is 12.8 kPa for predicting the presence of cirrhosis (AUC 0.789, 95% CI: 0.727–0.834, *p* < 0.001; Se 79.0%, Sp 65.5%, PPV 50.4%, NPV 87.5%, LR^+^ 2.3, LR^−^ 0.3) (Fig. 3).

### Diagnostic performance of INR for predicting PHLF

**Fig. 4:**
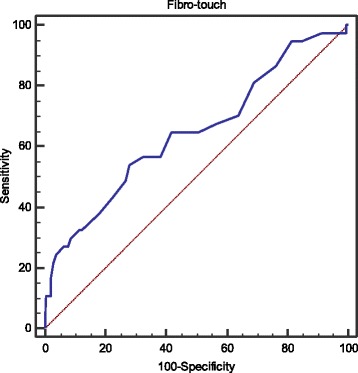
ROC analysis of INR for predicting PHLF

The optimal cutoff value of INR is 1.0 for predicting PHLF (AUC 0.646, 95% CI: 0.583–0.706, *p* < 0.001; Se 54.1%, Sp 71.9%, PPV 25.3%, NPV 89.9%, LR^+^ 1.9, LR^−^ 0.6) (Fig. 4).

### Diagnostic performance of the LSM-INR index for predicting PHLF

**Fig. 5:**
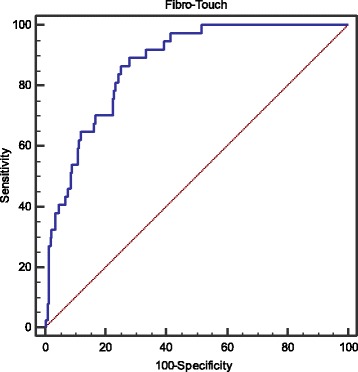
ROC analysis of model as the LSM-INR (LFM) index for predicting PHLF

The diagnostic performance and corresponding ROC curves of the LSM-INR index are shown in Fig. 5. The optimal cutoff value of the LSM-INR index is −1.9 for predicting the presence of PHLF (AUC 0.865, 95% CI: 0.822–0.909, *p* < 0.001; Se 86.5%, Sp 74.8%, PPV 37.6%, NPV 96.9%, LR^+^ 3.4 LR^−^ 0.2).

## Discussion

Surgical resection is the first-line therapeutic option for early HCC [21]. However, insufficient FHR may result in postoperative complications and even PHLF (4), which is a major cause of postoperative morbidity and mortality after elective hepatic resection [22, 23].

Liver resection for HCC patients with chronic liver diseases still carries higher risk of PHLF than normal liver resection [22, 24].To the best of our knowledge, this is the first published study on the effectiveness of LSM measurement in predicting PHLF in patients with hepatitis B related hepatocellular carcinoma since previous studies only suggested a potential role of LSM in predicting post-resection hepatic insufficiency. Furthermore, in those previous studies [10, 25], the background hepatic conditions causing HCC showed a great variability and included HCV, alcoholic and nonalcoholic steatohepatitis in addition to HBV. The baseline characteristics of patients differed by studies and it may result in a selection bias.

In this study, we used a new generation transient elastography, FibroTouch® for liver stiffness measurement (LSM) [9, 26], which has enhanced 2D–image-guided positioning function. It’s particularly advantageous for precise preoperative examination, because the examiners are able to set the the region of interest(ROI) in the non-tumor area. It may also explain why the optimal cut-off LSM value (14.0 Kpa) in this study is lower than that in previous studies [10, 12]. Although the conclusions of their studies were similar to that of this study, some differences have to be pointed out. First, although the study population was very similar to ours, the background of the population was not homogenous. Secondly, their definition of PHLF was only based on postoperative serum bilirubin levels, which configures a very high risk of irreversible PHLF (5 mg/dL for more than 5 days post operation) [27]. Perhaps for this reason, their LSM cutoff was higher and may miss milder grades of PHLF [25].

Using the calculated cutoff value of 14.0 Kpa, LSM had high specificity and negative predictive value for predicting PHLF. The value in liver failure group was significantly higher than that in non-liver failure group (21.4 Kpa vs. 12.7Kpa). It implied that FHR may become worse with the increase of LSM value. While the effectiveness of LSM for diagnosing cirrhosis is not high (AUC = 0.78, 95% confidence interval: 0.727–0.834), it may be confounded by the enrollment of patients with Child-Pugh Class A. Precisely because of this reason, one interesting result of this study is that the degree of resection is not a significant predictor of PHLF.

In our study, HBV DNA showed no statistical differences, which is not in line with a previous report [28], and a possible explanation is the inclusion of patients with HBV DNA higher than 10^4^ IU/ml. For such patients, we will first use antiviral drugs to control the amount of HBV, and then choose surgery. Univariate analysis showed that platelet count, LSM and others were significant prognostic factors for PHLF. This is consistent with the previous finding of risk factors for postoperative complications [29].

We must acknowledge this study has several potential limitations. Compared to similar articles, LSM was significantly better than ICG-15 and MELD score in the prediction of postoperative complications [10, 12], we do not routinely use ICG-15 or MELD to assess liver function. The number of the outcome of PHLF was relatively small, and further acquisition of cases and the external validation should be accomplished in the future. Second, our analysis did not include other variables that may affect the outcomes of surgery, such as the resected liver volume which is closely correlated with the functional liver reserve and postoperative results [11]. A well-designed, well-controlled, randomized study of a large population is required.

## Conclusions

In summary, our study showed that the preoperative LSM is a valid tool for surgeons in making therapeutic decisions in patients with hepatitis B related hepatocellular carcinoma. We also established a new index, LSM-INR index, which quantitatively evaluated the risk of the INR and LSM that should be useful for surgeons in making therapeutic decisions in patients with hepatitis B related hepatocellular carcinoma before hepatectomy.

## Abbreviations

AUC: The area under the ROC curve
EHBH: Eastern Hepatobiliary Surgery Hospital
FHR: Function of hepatic reserve
HBsAg: Hepatitis B surface antigen
HCC: Hepatocellular carcinoma
ICC: Intrahepatic cholangiocarcinoma
LR^−^: Negative likelihood ratio
LR^+^: Positive likelihood ratio
LSM: Liver stiffness measurements
NPV: Negative predictive values
PHLF: Postoperative liver failure
PPV: Positive predictive values
ROC: Receiver operating characteristic
Se: Sensitivity
Sp: Specificity
TACE: Transhepatic arterial chem otherapy and embolization
TE: Transient elastography
